# Unmasking Brugada Syndrome With Oral Propafenone in a Resource-Limited Setting: A Case Report

**DOI:** 10.7759/cureus.116494

**Published:** 2026-09-18

**Authors:** Luisa Fernanda Jaramillo Astudillo, Guillermo Adrian Navas Moreira, Patricia Delgado Cedeño, Cesar Joao Tumbaco Mite, Estefania Carolina Arteaga Herrera

**Affiliations:** 1 Clinical Research, Instituto Ecuatoriano del Corazon (IECOREC), Guayaquil, ECU

**Keywords:** brugada syndrome, oral propafenone, pharmacological provocation, resource-limited setting, unexplained syncope

## Abstract

Brugada syndrome (BrS) is a cardiac channelopathy predisposing patients to malignant ventricular arrhythmias and sudden cardiac death (SCD) in the absence of structural heart disease. The diagnosis requires documentation of a type 1 Brugada electrocardiographic pattern, which can be unmasked using sodium channel-blocking agents in symptomatic patients presenting with non-diagnostic baseline patterns. We report a case of a 24-year-old male patient with a history of unexplained syncope who exhibited a non-diagnostic type 2 Brugada pattern on a baseline electrocardiogram (ECG). Due to the local unavailability of standard intravenous antiarrhythmics, a pharmacological provocation test was conducted using a single 600 mg oral dose of propafenone. Under continuous cardiac monitoring, the patient safely developed a diagnostic type 1 "coved" electrocardiographic pattern starting at the third hour, confirming the diagnosis of BrS. The ECG returned to its baseline pattern eight hours after the initiation of the test, and no cardiovascular complications occurred. This case highlights the clinical utility and safety of oral propafenone as an effective alternative diagnostic challenge for unmasking BrS in resource-limited environments. Furthermore, it emphasizes the necessity of utilizing extended monitoring protocols due to the specific pharmacokinetic profile and delayed onset of action of oral agents.

## Introduction

Brugada syndrome (BrS) is an inherited cardiac channelopathy that increases the risk of sudden cardiac death (SCD) and malignant ventricular arrhythmias, despite the absence of underlying structural heart disease [1]. Definitive diagnosis is established by the presence of a type 1 Brugada electrocardiographic pattern, which may occur spontaneously or be unmasked via sodium channel-blocking agents. Given the intermittent nature of these electrocardiographic manifestations, pharmacological provocation is recommended for symptomatic patients who present with non-diagnostic baseline patterns [1,2].

Intravenous agents such as ajmaline or flecainide are the standard drugs for diagnostic challenge [3]; however, their availability is limited in many resource-constrained settings. Propafenone, an oral class IC antiarrhythmic with sodium channel-blocking properties, has been reported to unmask Brugada patterns and may represent a practical alternative [2,4]. We report a case in which oral propafenone successfully revealed a diagnostic type 1 Brugada pattern in a young patient with syncope, highlighting its potential utility in settings with limited access to standard agents.

## Case presentation

A 24-year-old Hispanic male patient presented to the emergency department complaining of palpitations, chest pain, and dyspnea on exertion. These symptoms were precipitated by an emotional stressor three days prior, culminating in a syncopal episode lasting several seconds. The patient’s medical history was notable for a previous uninvestigated syncopal event nine years earlier while running. He denied any history of chronic systemic illness, and there was no family history of SCD, cardiac arrest, or documented arrhythmias. Upon admission, the patient was hemodynamically stable, and the physical examination was unremarkable.

Initial laboratory evaluations, including high-sensitivity cardiac troponins, NT-proBNP, and routine metabolic panels, were within normal limits. A 12-lead electrocardiogram (ECG) performed at admission revealed a type 2 Brugada pattern characterized by a saddleback morphology in the precordial leads (Figure 1A). Transthoracic echocardiography demonstrated preserved biventricular function with no evidence of structural or anatomical abnormalities.

**Figure 1:**
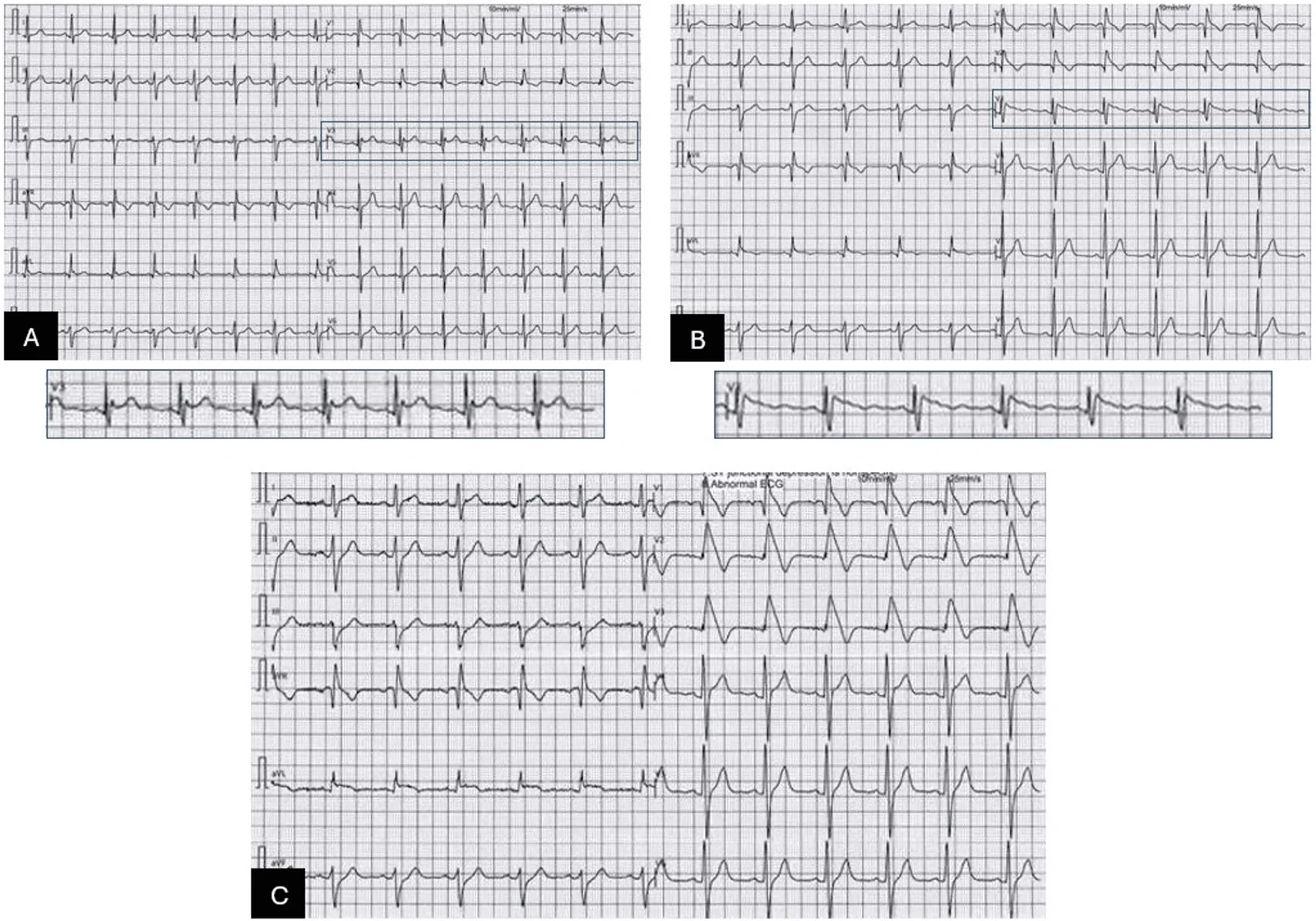
Serial 12-lead ECGs documenting the evolution of Brugada pattern morphology during oral propafenone drug challenge. (A) Baseline ECG at admission showing a type 2 Brugada pattern characterized by a saddleback ST-segment morphology in the right precordial leads; the inset below provides a magnified view of lead V3 (blue box) highlighting the ST-segment elevation. (B) ECG obtained two hours after oral propafenone administration, with the corresponding inset showing the persistence of the morphology. (C) ECG obtained four hours post-administration, demonstrating conversion to a diagnostic type 1 Brugada pattern in the right precordial leads with ≥2 mm coved ST-segment elevation followed by a negative T wave. ECG, electrocardiogram.

During hospitalization, the patient remained hemodynamically stable and asymptomatic. Given the baseline type 2 electrocardiographic pattern and the history of syncope, a pharmacological provocation test (drug challenge) was indicated to rule out or confirm a type 1 Brugada pattern. Due to national pharmacological regulations and the lack of importation of intravenous class IA or IC antiarrhythmics (e.g., ajmaline or flecainide) into the local healthcare system, an oral class IC sodium channel blocker (propafenone) was utilized as an alternative diagnostic agent [5].

The provocation test was performed in the coronary care unit under continuous cardiac monitoring, with external defibrillator pads and backup pacing capabilities in place. A single 600-mg oral dose of propafenone was administered. Serial ECGs were obtained every five minutes during the first hour, every 15 minutes during the second hour, and every 30 minutes up to the fourth hour. The patient did not report any symptoms.

During the first two hours, the ECG maintained the saddleback pattern in lead V3 and a right bundle branch block-like pattern in V2 (Figure 1B). However, from the third hour onwards, a shark-fin pattern became evident in leads V1, V2, and V3. This was distinctly observed at the fourth hour, featuring an initial ST-segment elevation of ≥2 mm with a rectilinear descent toward the isoelectric baseline, followed by a negative, symmetric T wave, forming a “coved” appearance (Figure 1C). The test was concluded, and an ECG performed eight hours after the initiation of the challenge demonstrated a return to the initial type 2 Brugada pattern.

Based on the clinical history of unexplained syncope and the positive pharmacological challenge unmasking a type 1 pattern, the patient met the criteria for BrS. To definitively exclude structural mimics, such as arrhythmogenic right ventricular cardiomyopathy (ARVC), a cardiac magnetic resonance (CMR) scan was performed, focusing on the right ventricular outflow tract (RVOT); findings were normal (Figure 2). No cardiovascular symptoms or complications occurred during the procedure or the 24-hour follow-up.

**Figure 2:**
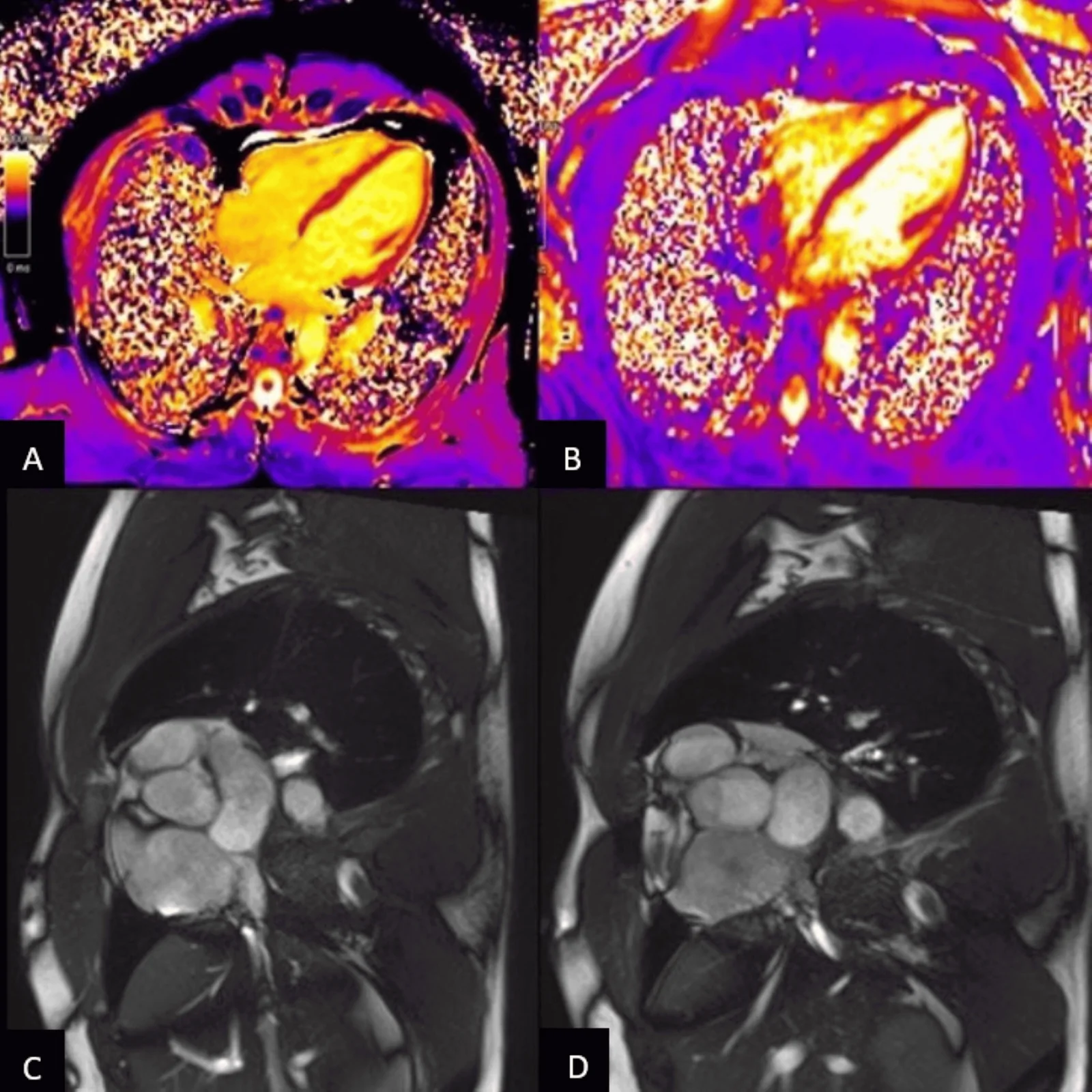
CMR imaging to definitively exclude structural heart disease. (A, B) Four-chamber views with tissue characterization mapping demonstrating normal biventricular size, preserved myocardial thickness, and the absence of abnormal tissue infiltration (such as myocardial fibrosis) suggestive of ARVC. (C, D) Morphological views focusing on the RVOT during different phases of the cardiac cycle demonstrating normal anatomy without evidence of dilation, aneurysms, or regional wall motion abnormalities. CMR, cardiac magnetic resonance; ARVC, arrhythmogenic right ventricular cardiomyopathy; RVOT, right ventricular outflow tract.

## Discussion

BrS is an inherited channelopathy associated with an increased risk of ventricular fibrillation (VF) and SCD in structurally normal hearts. Epidemiologically, BrS is estimated to be responsible for 4% to 12% of all SCD cases and up to 20% of cases in patients without structural heart defects [6]. The prevalence varies significantly worldwide, affecting approximately 0.5 per 1,000 individuals globally but reaching endemic levels in Southeast Asian populations [6]. A striking clinical feature is its strong male predominance, with a male-to-female ratio as high as 8:1, suggesting a pathophysiological mechanism involving hormones [3,4]. Clinical manifestations typically peak in the third or fourth decade of life, closely aligning with the profile of our 24-year-old male patient [7].

The syndrome is characterized by a distinct coved-type ST-segment elevation in the right precordial leads (type 1 pattern). However, clinical diagnosis is often complex because the electrocardiographic pattern can be dynamic, intermittent, or concealed. Recent literature emphasizes that while genetic factors, such as SCN5A mutations, play a role in approximately 20-30% of cases, the phenotypic expression is variable [8]. This variability makes the integration of clinical history and electrocardiographic findings crucial for accurate diagnosis [3]. Furthermore, the underlying mechanism remains a subject of debate between the repolarization hypothesis (transmural dispersion of repolarization) and the depolarization hypothesis (conduction slowing in the RVOT). Recent evidence also suggests that microscopic structural alterations, such as fibrosis or inflammation, may contribute to the substrate, challenging the traditional view of a purely functional channelopathy [4].

The cornerstone of BrS diagnosis is the documentation of the type 1 electrocardiographic pattern, either spontaneously or induced by sodium channel-blocking agents. Definitive diagnosis requires the integration of this pattern with specific clinical criteria. These criteria include documented VF or polymorphic ventricular tachycardia (VT), a family history of SCD at <45 years of age, coved-type ECGs in family members, inducibility of VT with programmed electrical stimulation, unexplained syncope, or nocturnal agonal respiration [2]. In patients presenting a baseline type 2 (saddleback) pattern alongside highly suggestive clinical criteria, such as the unexplained syncope observed in our patient, current guidelines strongly recommend a pharmacological provocation test to confirm the diagnosis [7].

The standard agents for unmasking BrS are class IC antiarrhythmic drugs. The 2022 European Society of Cardiology (ESC) Guidelines for the management of ventricular arrhythmias recommend the use of ajmaline or flecainide due to their short half-lives and high diagnostic yield [9]. However, the availability of intravenous forms of these drugs is often limited in specific regions, including parts of Latin America. In this context, oral propafenone has been identified as a viable alternative diagnostic agent. Although less commonly used than ajmaline, propafenone acts as a potent sodium channel blocker capable of revealing the arrhythmogenic substrate in the RVOT [8].

To contextualize the novelty of the present case, we conducted a structured literature search in PubMed using the terms “Brugada syndrome AND propafenone,” which yielded only 33 results at the time of manuscript preparation. Previous reports have predominantly described the unmasking of Brugada electrocardiographic patterns associated with propafenone in the setting of overdose or toxicity, or incidentally during antiarrhythmic treatment, particularly for atrial arrhythmias [10]. To the best of our knowledge, this is the first reported case in Ecuador, and one of the few in Latin America, in which oral propafenone was intentionally administered to unmask a diagnostic type 1 Brugada electrocardiographic pattern.

In the reported case, the use of a high-dose oral propafenone challenge (600 mg) successfully converted the inconclusive type 2 pattern into a diagnostic type 1 pattern after four hours. This aligns with previous reports where propafenone, sometimes identified in the context of accidental overdose or toxicity, has demonstrated the capacity to induce distinct Brugada-like electrocardiographic changes [9,11]. The delayed onset of the type 1 pattern in our patient (three to four hours) highlights the pharmacokinetic differences between oral propafenone and intravenous agents, requiring prolonged monitoring protocols to ensure diagnostic evidence and patient safety [4].

Nevertheless, this approach should be interpreted cautiously. The sensitivity, specificity, and negative predictive value of oral propafenone as a diagnostic provocative agent for BrS have not been systematically established. Unlike standardized intravenous protocols using sodium channel blockers such as ajmaline or flecainide, oral propafenone is subject to variability in gastrointestinal absorption, hepatic first-pass metabolism, and a later peak plasma concentration. Consequently, a negative provocative test with oral propafenone cannot be considered conclusive and should not be used to exclude BrS. Therefore, the present case illustrates a pragmatic, non-standardized diagnostic strategy in a resource-limited setting rather than a validated substitute for conventional pharmacological provocation testing [7,9].

Risk stratification remains a pivotal aspect of BrS management. Our patient presented a history of syncope, which is a recognized independent marker for a high risk of arrhythmic events. The integration of clinical symptoms, such as syncope, with a drug-induced type 1 pattern significantly increases the Shanghai Score, solidifying the diagnosis and guiding the need for aggressive management, including the consideration of an implantable cardioverter-defibrillator (ICD) [12]. Additionally, the literature suggests that specific electrocardiographic markers, such as fragmented QRS (f-QRS) and a prolonged Tpeak-Tend interval, may serve as non-invasive predictors of arrhythmic events, providing a stronger risk profile beyond clinical symptoms alone [8,13].

Finally, long-term management extends beyond device therapy. Patients must be educated to avoid specific arrhythmogenic drugs, limit excessive alcohol intake, and treat fever aggressively with antipyretics, as these are potent triggers for lethal arrhythmias [1,8]. Regarding specific therapies, ICD implantation should be considered in patients with a history of cardiac arrest, documented sustained ventricular arrhythmias, or a spontaneous type 1 pattern with arrhythmic syncope. Quinidine may also be considered in selected patients with recurrent ventricular arrhythmias, multiple appropriate ICD therapies, or in those for whom ICD implantation is indicated but cannot be performed or is declined [9].

If our patient had not developed a type 1 pattern after oral propafenone administration, BrS would have remained within the differential diagnosis because of the baseline type 2 pattern and history of unexplained syncope. In that scenario, continued clinical and ECG follow-up would have been appropriate, with a conventional sodium channel blocker challenge performed when available, rather than considering a negative oral propafenone response sufficient to exclude the diagnosis.

This case underscores the utility of adapting diagnostic protocols to local resource availability without compromising clinical rigor, ensuring that young patients with potentially lethal channelopathies are correctly identified and managed.

## Conclusions

BrS is a clinical entity characterized by specific electrocardiographic changes in the right precordial leads and a significant predisposition to SCD in the absence of structural heart disease. Given the dynamic nature of electrocardiographic abnormalities, which may normalize temporarily, pharmacological provocation remains the gold standard for diagnosis in uncertain cases. Our case illustrates how oral propafenone may represent an alternative diagnostic strategy for unmasking a type 1 Brugada pattern when conventional intravenous sodium channel blockers are unavailable. Although propafenone has previously been reported to unmask Brugada-like electrocardiographic changes, its intentional use as a diagnostic provocative strategy remains uncommon. However, oral propafenone should not be considered an established substitute for conventional provocative testing, as its diagnostic sensitivity and specificity have not been systematically validated. Therefore, failure to induce a type 1 pattern cannot reliably exclude BrS. Ultimately, this case highlights the importance of adapting diagnostic strategies to resource availability while maintaining appropriate safety measures. It also underscores the crucial need to recognize high-risk clinical features, particularly arrhythmic syncope, to ensure the timely implementation of life-saving therapies, such as ICD implantation.
